# Penile reconstruction: An up-to-date review of the literature

**DOI:** 10.1080/2090598X.2021.1957410

**Published:** 2021-07-26

**Authors:** Nicholas Ottaiano, Joshua Pincus, Jacob Tannenbaum, Omar Dawood, Omer Raheem

**Affiliations:** Department of Urology, Tulane University School of Medicine, New Orleans, LA, USA

**Keywords:** Penile reconstruction, Peyronie’s disease, penile trauma, phalloplasty, penile transplant, inflatable penile prosthesis

## Abstract

**Objective:**

: To review the literature on adult penile reconstruction due to Peyronie’s disease, trauma and cosmesis, while emphasising specific surgical techniques and procedures such as phalloplasty, radial forearm free flap reconstruction, and penile transplant.

**Methods:**

: A comprehensive review of the literature for the years 1992–2020 of the PubMed and SpringerLink databases was performed to identify articles on penile reconstruction. Search terms included ‘penile reconstruction’, ‘penile trauma’, ‘phalloplasty’, ‘penile transplant’, and ‘treatment of Peyronie’s’. Relevant articles were selected. All included studies were performed on adults and written in English.

**Results:**

: We were able to identify 46 papers from PubMed and SpringerLink that included the research terms. From these, we included technical details of procedures and gleaned photographs of their works. Additionally, we included photographs from our institution’s own plication surgery cases.

**Conclusions:**

: The field of adult penile reconstruction is performed for a plethora of reasons. From cosmetic to urgent and from routine to complex, it is most certainly a growing subset of Urology that plays a vital role for the men who need it. To our knowledge, this is the most up-to-date review of adult penile reconstruction.

## Introduction

The area of adult penile reconstruction is incredibly vast. Ranging from more common procedures, such as penile prosthetic surgery, to some of the most complex cases known to the medical literature. Penile reconstruction is performed for many reasons, including emergent, functional, and cosmetic. The field of men’s health has undergone rapid expansion over the last two decades; however, research regarding the subset of penile reconstruction remains largely scattered and at times, sparse. The present systematic review serves an important role: to gather the relevant data to present our findings in one communication. In the present review, we explore adult penile reconstruction from the most up-to-date perspective.

Structural deformities of the penis, such as those seen in Peyronie’s disease (PD), often necessitate surgical intervention, as surgery has been proven to be the most reliable method [1]. With minimal medical treatment available, the decision as to which type of surgical procedure is patient dependent. Various factors such as degree of curvature and patient anatomy come into play, all of which must be considered to ensure the best possible outcome [1,2]. Whether the deformity be congenital or traumatic, penile reconstruction shares the common goal of creating a cosmetically appealing phallus that is capable of both micturition and sexual intercourse. In the case of severe trauma, surgical repair is almost inevitable and must be undertaken promptly in hopes of preserving as much viable tissue as possible due to the unique features of penile tissue not found elsewhere on the body. When primary repair is not feasible, the use of skin grafts from locations such as the forearm and thigh can facilitate reconstruction [3].

We begin with some of the more well-known conditions requiring penile reconstruction, including PD and penile trauma, exploring different techniques in plication and exploratory surgery. We then transition to discuss some of the most complex penile reconstructive surgeries ever attempted, namely penile transplantation and forearm flap reconstruction. We aim to give the reader a general purview of penile reconstructive surgery, all the while exploring each topic in technical detail. We hope this up-to-date review on penile reconstruction will serve all those seeking to gain a better knowledge in this complex, growing subset of urology.

## Methods

A systematic review of the literature was performed in September 2020 following the Preferred Reporting Items for Systematic Reviews and Meta-Analyses (PRISMA) guidelines [4]. An extensive search was carried out using the databases of PubMed and SpringerLink using various combinations of keywords such as the following: ‘penile reconstruction,’ ‘penile trauma,’ ‘phalloplasty,’ ‘penile transplant,’ and ‘treatment of Peyronie’s.’ Attention was given to studies written in the context of urology with an emphasis on penile reconstruction. Only English-language articles dated within 2007–2020 were included in the search, with the exception of 10 manually selected articles due to the limited availability of articles and the unique subject matter. All studies included were performed in adults and had an abstract available.

## Results

**Figure 1.**
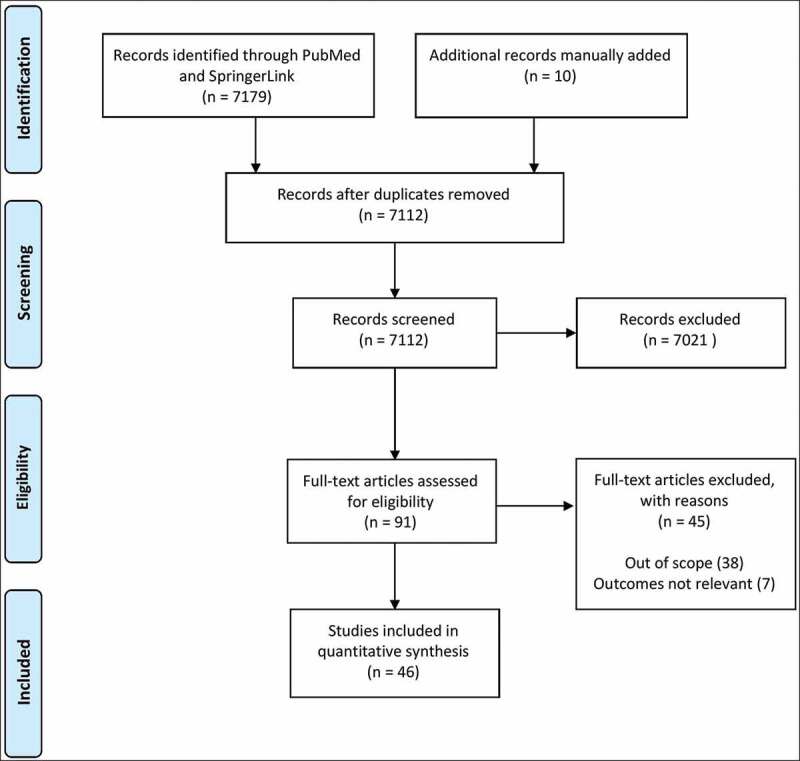
Flow chart of study selection according to PRISMA guidelines [4]

After the initial search of the literature, 7179 articles were identified. Again, an additional 10 records were added manually due to the topics being a combined interest of both urology and plastic surgery. After removing duplicates, 7112 records remained for screening. A total of 7021 were excluded after screening of titles and abstracts. Of the 91 full-text articles assessed for eligibility, 38 were excluded for being out of the scope along with an additional seven for having outcomes not relevant to this review. The remaining 46 articles were selected as eligible for the present review (Figure 1).

## Peyronie’s disease

Peyronie’s disease (PD) is an acquired condition that often necessitates the need for urological management by both surgical and non-surgical means. This is a disorder of the tunica albuginea resulting in fibrous scar tissue causing curvature of the penis and, oftentimes, painful erections. The prevalence has been estimated to be ~9%, affecting men of all ages including those in their teenage years [5,6].

Surgical reconstruction remains the ‘gold standard’ treatment option in those with PD. Available procedures include penile plication, penile grafting, and inflatable penile prosthesis (IPP), with the goal being both to improve sexual function and create a more cosmetically appealing appearance. On the less invasive end of the spectrum, options such as penile traction therapy, intralesional injections of collagenase *Clostridium histolyticum* (CCh), and stem cell therapy are viable alternatives for those wishing to avoid surgery and its possible complications [1,7].

**Figure 2.**
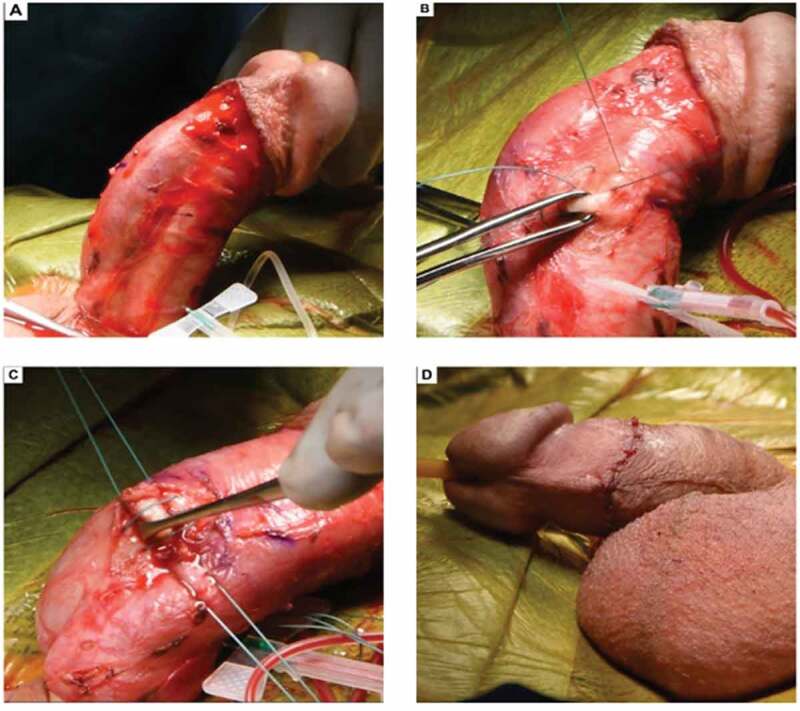
Penile plication for correction of PD. (Photographs used with permission from Dr Wayne Hellstrom, Tulane University Department of Urology, New Orleans, LA, USA)

In men with curvatures of <60°, penile plication is often the best suited strategy, as it is associated with low morbidity and high efficacy. Various techniques have been described dating back to 1965 with the original Nesbit procedure, although several modifications have since surfaced. Essentially, a small ellipsoid-shaped portion opposite to that of the most prominent area of fibrosed tunica albuginea is excised. The remaining defect is then closed with nonabsorbable suture resulting in a significant reduction in penile curvature [2] (Figure 2).

Depending on the technique used, clinical outcomes include length loss, curvature re-occurrence, penile bruising, and temporary sensory change in the glans. Overall, penile plication has been associated with overall patient satisfaction (67–100%) and high success rates (79–100%) [1]. Estimated stretched penile length loss is around 0–2.5 cm, depending on the technique used and the degree of curvature [8].

For those with curvatures in the severe range (>60°), penile lengthening surgery with graft reconstruction is recommended. Graft surgery technique is typically unchanged amongst varying methods with dissection of the neurovascular bundle sometimes required depending on the anatomical location of the plaque to ensure curvature does not re-occur. The graft is inserted and secured to the tunica albuginea after ensuring the entirety of the plaque is removed. The choice of graft material and technique depends on a multitude of factors such as patient preference, specific factors to that of the patient’s disease, surgeon’s experience, and availability of graft material. Clinical outcomes include temporary sensation loss to the glans, haematoma, penile bruising, harvest-site complications, graft contracture with length loss, curvature re-occurrence and erectile dysfunction (ED) [1].

Patients with ED or those who fail to respond to alternative therapies for their ED would be candidates that would stand to benefit from an IPP. In men with curvatures of <30°, satisfactory results can often be obtained simply from insertion of an IPP alone. However, those with a curvature of >30° frequently need further corrective measures to achieve a desired result after placement of an IPP. Generally, an IPP plus the addition of either penile plication, grafting, or manual modelling can be undertaken at the time of IPP surgery to reduce significant curvature. Adverse clinical outcomes can include altered glans sensation, infection, haematoma, prosthesis malfunction, glans necrosis, and graft contracture with recurrent curvature [1].

## Trauma

**Figure 3.**
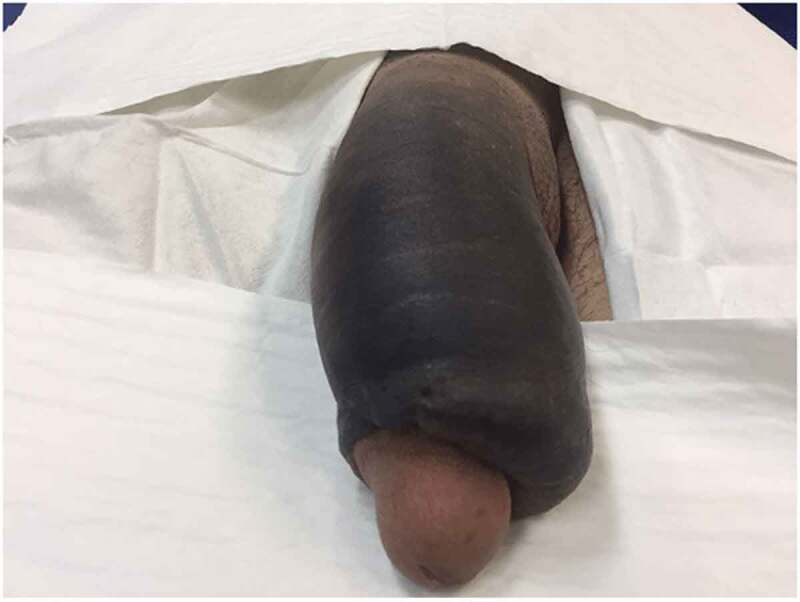
Penile fracture classically presenting with an ‘eggplant deformity’ where haemorrhage extends beyond the tunica albuginea resulting in swelling and ecchymosis. (Photograph courtesy of Mirzazadeh et al. [12])

Penile trauma can occur due to a variety of reasons. Unfortunately, the incidence is under reported in the literature due to failure to seek medical attention for psychological and ethical reasons [9]. Penile fracture (PF) is a urological emergency that occurs when there is a tear in the tunica albuginea and, occasionally, rupture of the corpus cavernosum. The most common aetiology of PF in the USA is due to sexual intercourse. This is primarily a diagnosis made on history and physical examination alone, although imaging in the form of ultrasonography can be of use if uncertainty remains [10]. Patients typically report an inciting event resulting in an audible cracking followed promptly by pain. Physical examination often shows penile deformity classically described as an ‘eggplant deformity’, ecchymosis, and oedema [11] (Figure 3 [12]).

Immediate surgical fixation is currently the recommended option to best explore and repair the tunica defect. The overarching opinion for this approach is based upon achieving the quickest recovery and satisfactory cosmetic results. Postoperative complications include painful intercourse, pain with erections, wound oedema, wound infection, skin necrosis, and wound dehiscence [10]. When comparing those who elected to undergo immediate surgical fixation for PF to those who opted for conservative treatment, it was found that the overall complication rates were 20.6% and 46.4%, respectively [13].

Penile soft tissue injury is another common means of injury. These injuries typically occur by means of strangulation or entrapment. Strangulation is a form of compartment syndrome frequently occurring in adults for means of prolonging erections for sexual gratification. Urgent treatment is required to avoid permanent damage [14]. The treatment of choice often depends on a multitude of factors such as material of the strangulating object and availability of resources. Commonly used techniques are use of cutting devices, string and aspiration method, and degloving operation. The first step in treatment typically starts with application of a lubricant with an attempt of manual removal. This process is usually performed concurrently with the string and aspiration method, where blood is aspirated from the corpora with an 18-G needle to achieve decompression followed by tightly winding string distal to the ringed object in hopes of sliding the ring over the string. If this fails, more drastic measures are taken by means of intraoperative bone or wire cutters and, possibly, power drilling machinery. After object removal, urethral inspection and possible skin grafting may be required depending on the extent of injury [14,15].

Penile entrapment is often seen in young boys in which the foreskin becomes caught within the teeth of a zipper. Simply cutting the material between the trapped foreskin and zipper teeth is usually adequate. A more complicated zipper removal requiring careful cutting via wire or bone cutters may be necessary in cases of entrapment in which the foreskin was trapped within the zipper slider [9].

Penetrating trauma is much more common in the military setting compared to that of the general public, accounting for 14.8% and 0.57% of all patients presenting with external genital trauma, respectively [16,17]. Ballistic wounds from projectiles can cause penetrating injury to the penis. The damaged area can be classified into zones to better aid in understanding of how each area of tissue will respond to injury. The primary tract the penetrating projectile leaves behind from coming into direct contact with tissue by directly piercing through is referred to as zone 1. Another temporary zone created from the shearing of tissue via energy waves from the projectile resulting in blood vessel rupture, muscle damage, and a zone of haemorrhage adjacent to zone 1 is designated as zone 2. Lastly, zone 3 extends further into the tissue because of the dispersion of shock waves. The recovery of tissues in zones 2 and 3 is variable, as injury progression often takes days to progress. This variability is especially pronounced when immediate debridement is prolonged after initial injury [17].

The primary treatment approach to penetrating trauma is aggressive surgical debridement and preservation of viable tissue, which often times must be done over the course of multiple procedures. The use of negative pressure wound therapy has also found its way into both the military and civilian world. The use of such devices can help reduce multiple painful dressing changes and better accommodate the irregular contour of the male external genitalia without compromising blood flow [17,18].

## Penile transplant

The first penile transplant was performed in 2006 in Guangzhou, China. Very few of these procedures have been documented in the medical literature, of which they have produced mixed results.

The goals of penile transplantation for recipients are cosmetic appearance, the ability to void standing up, and erectile function [19]. While typically not the usual first line of treatment, penile transplant can be indicated in situations in which current methods of reconstruction have failed to adequately restore the normal anatomy and function of the penis. Furthermore, within the wounded military population, both the radial forearm free flap (RFFF) and the pedicled anterolateral thigh flap (ALTF) for neophalloplasty may not be viable options to serve for reconstruction in patients who may have simultaneous limb damage [20].

The surgical technique used among transplant recipients is highly variable and based on a multitude of factors such as both donor and recipient anatomies, skill level of the surgeon, and availability of resources. The overarching concern when performing a penile transplant is how to best vascularise the allograft. Some critical learning points discovered during initial cadaveric trials included findings such as the anastomoses of the cavernosal vessels supplying the majority of the corpora, which can potentially improve postoperative erectile function, possibly avoiding the need for a future IPP. Another finding was the use of the external pudendal artery may reduce the complication of penile shaft skin necrosis, as these early trials showed the vessel to provide the vast majority of the area’s vascularisation [20].

Beyond the surgical complexity comes the added burden of lifelong immunosuppressive regimens that, like all transplant procedures, pose an additional risk. However, despite these risks, evidence exists that show possible benefits from certain immunosuppressants. Tacrolimus, a commonly used immunosuppressant in the post-transplantation setting, demonstrated to have a stark contrast when compared to cyclosporine. Tacrolimus did not impair smooth muscle relaxation and was shown to have less of an adverse effect on erectile function. In fact, some research even demonstrates tacrolimus to increase nerve regeneration and expedite recovery of erectile function. A neuroprotective and neurotrophic effect has also been demonstrated in rodent models with cavernosal nerve injuries. Of note, should phosphodiesterase type 5 inhibitors be needed postoperatively to achieve adequate erections, no adverse interactions between either cyclosporine or tacrolimus have been recorded [21,22]. Not all immunosuppressants are created equally though. For example, following two retrospective analyses of patient’s status after renal transplantation, a correlation between cyclosporine and ED was discovered. This effect could in part be attributed to the drug’s vascular endothelial cell dysfunction or inhibition of nitric oxide‐mediated smooth muscle relaxation [21].

## Phalloplasty

Phalloplasty is the surgical creation of a penis-like structure. The first successful phalloplasty was reported in 1936 using rib cartilage and an abdominal flap [23]. Over time, there have been many advances in flap techniques and neophallus designs [24]. Typically, two operative teams work simultaneously; the plastic surgery team harvests the donor site flap while the urological team prepares for and then places the flap as a neophallus [25]. Phalloplasty can be performed in a single procedure, but more commonly it is performed as staged procedures that can be done months apart [26]. There are various indications for phalloplasty, including penile insufficiency in cis-males and female-to-male gender reassignment surgery. In cis-males, penile insufficiency can be secondary to congenital disorders, surgical or traumatic amputation of the penis, penile fracture, and Fournier’s gangrene [23,25,27]. Relative contraindications include a body mass index of >35 kg/m^2^ and truncal obesity due to added postoperative risk and increased thickness of donor sites [26]. Additionally, compliance and health literacy should be assessed, as there are frequent postoperative appointments and enormous health and lifestyle consequences [26].

**Figure 4.**
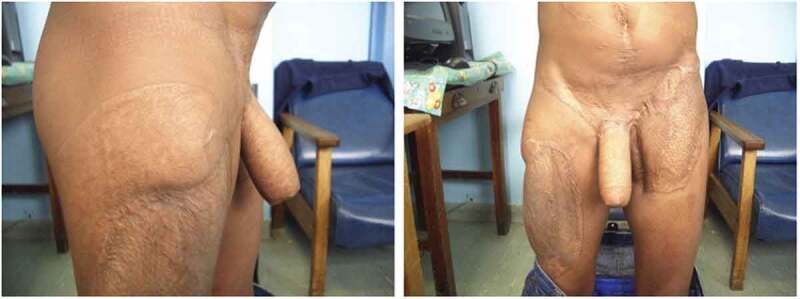
Postoperative phalloplasty results in a patient left without testis or penis after a blast injury. (Photographs courtesy of Descamps et al. [30])

Goals of phalloplasty include the creation of an aesthetically penis-like structure with sensation, the ability to have an erection, and intact micturition [26]. In cis-men, compared to trans-men, phalloplasty can incorporate native genital tissue to better improve orgasmic function [25]. The vast majority of patients prefer for the neophallus to allow for voiding while standing and thus also require the construction of a neourethra [23,28]. Alternatively, if the patient accepts sitting urination as an outcome, a shaft-only phallus can be constructed without the need for urethral lengthening [26]. Another important component is the reconstruction of a glans, or glansplasty [24]. Glansplasty includes the creation of a coronal ridge and sulcus [29]. In paediatric patients, it is important to consider timing of surgery, as the neophallus does not grow in response to pubertal hormones as a penis would; generally, the creation of an adult-sized neophallus should be planned near the time of puberty [23] (Figure 4 [30]).

A major decision is the selection of a donor site. The donor site should have healthy skin that is thin and relatively hairless [26]. The most commonly used donor sites are the forearm and anterolateral thigh [26]. As we will discuss, the RFFF is the gold standard, and is the most commonly performed type [31]. RFFF achieves high patient satisfaction with cosmetic appearance and phallic urination [26]. However, RFFF does leave a large scar on the forearm that patients may find unappealing [26]. The ALTF is a good option for patients that prefer to hide the donor scar or want a longer phallus with more girth; however, this can often result in a disproportionately large or bulky neophallus [26,31]. An advantage to ALTF is that the anterolateral thigh skin is a better colour match to the perineum, which is especially important for patients with pigmented skin [30]. Less commonly used alternative donor sites include the ulnar forearm free flap, latissimus dorsi free flap, abdominal flap, and superficial circumflex iliac artery perforator flap [24,26,32,33]. There are also multiple neophallus designs. The shaft-only design does not include a neourethra [26]. The most popular design is a tube-within-a-tube, which involves two skin paddles rolled in opposite directions [26,33]. The composite design uses separate donor sites for the urethra and shaft, which can be useful for patients that want to minimise forearm scarring from RFFF but have unsuitable thigh skin for a pure ALTF [26]. A less common option is the vascularised shaft, grafted urethra design, which is the combination of a shaft-only phalloplasty and the construction of a urethra using a graft [26].

Outcomes of phalloplasty include a high rate of complications but also high rates of satisfaction. Urethral complications occur in one-third of all patients, but over three-quarters of patients are capable of voiding while standing [34]. Urethral complications are mainly fistulae and strictures, which are commonly the cause of revision surgery [31,34]. Fistulae commonly occur at points of anastomosis, while strictures occur at watershed areas of decreased blood supply [26,35]. Urethral complications can compromise quality of life and cause chronic infection, which can lead to sepsis and renal failure [35]. Flap complications occur in 10% of patients [34]. The most severe result is a full phallic loss, which has an estimated rate of 1.69% [26]. Partial phallic loss is more common and can lead to infection and the need for further surgical management [26]. Infection is prevalent due to proximity to the groin, genitals, and rectum [26]. Other common flap complications include haematoma and wound dehiscence [26]. Ultimately, patient satisfaction is reported as high (84–90%) [34,36]. Achievement of sexual function is varied, with reports between 61–100% [34,36].

## Radial forearm free flap reconstruction

**Figure 5.**
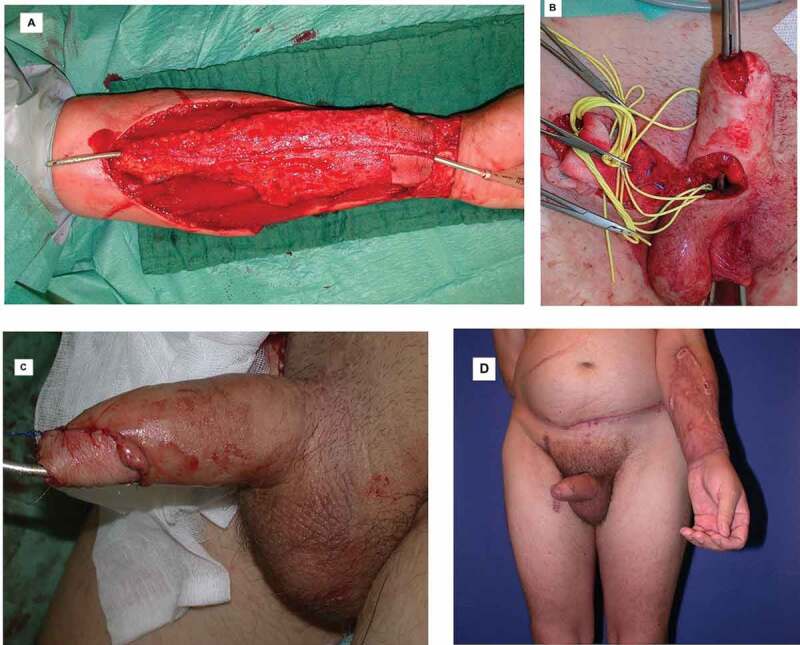
(A) RFFF tubed to form neourethra. (B) Phallus prior to insertion of RFFF. (C) Well-vascularised semierect after micro-anastomosis (D) Follow-up visit after successful RFFF and full-thickness graft harvested from left groin. (Photographs courtesy of Dabernig et al. [37])

A RFFF is a viable surgical option for penile reconstruction due to the predictable anatomy of the flap, pliable skin, and well-developed vessels [37] (Figure 5). The RFFF is harvested from the forearm and shaped to the phallus using the tube-within-a-tube technique wherein two skin paddles are rolled in opposing directions with a dermal vascular supply between the layers to supply the urethral skin paddle. An additional skin flap is then used to create a corona to mimic a circumcised glans. After anastomosing the urethra, the free flap is moved into place on the pubic area for the radial artery to be connected in an end-to-side fashion to the common femoral artery via microsurgical technique. The anastomosis of the venous drainage is also made microsurgically between the greater saphenous vein and the cephalic vein. Additionally, a cutaneous nerve, often the medial cutaneous nerve of the forearm, is connected to the ilioinguinal nerve to maintain protective sensation, while the dorsal penile nerve is connected to another nerve to achieve erogenous sensation [38].

The final stage of the RFFF is the implantation of an IPP to allow for penetrative intercourse. This step is typically performed after a year to allow for adequate sensory nerve recovery. Possible complications of RFFF include formation of a urethral fistula, stricture, complete flap loss, partial flap loss, distal necrosis, need for re-grafting, and decreased sensation. However, the primary pitfall of the RFFF is due to the forearm grafting site. In addition to the scar left from graft harvesting, other complications include cellulitis, compartment syndrome, decreased strength, paresthesias, and neuromas. Overall outcome and patient satisfaction were generally favourable, with some studies showing up to 97% patient satisfaction and up to 60% reported being able to partake in penetrative intercourse following insertion of an IPP [39].

## Cosmetic

Proposed and performed purposes for cosmetic penile surgery include buried penis disease with the goal to improve penile lengthening. Buried penis disease occurs most often in men who have become obese. Adult-acquired buried penis (AABP) is diagnosed by the accumulation of fat tissue thus affecting the lower abdominal skin and soft tissue advancing over the penis [40]. Consequently, this makes the penis decrease in length or become completely buried [41]. Patients with AABP typically present with poor sexual function including urinary dribbling, skin break, urethral strictures, mood disturbance, lichen sclerosis, and poor quality of life [42,43]. The first line of treatment is weight loss; however, this may not resolve AABP due to permanent fibrotic penile skin changes along with lymphoedema of the escutcheon. Most patients will need surgery for long-term management of AABP.

Preoperative considerations are important due to most patients being obese. As such, they present with multiple comorbidities and typically with diabetes mellitus [44]. Evaluation of haemoglobin A1c is critical for proper healing to occur, with the patient demonstrating their ability to maintain glucose control. Consideration of a possible urethral stricture should be evaluated, as a recent study of 42 patients [45] documented that ~31% of patients had anterior urethral strictures that needed to be managed prior to AABP surgery. In order to increase success of graft placement during AABP procedures, a Kulkarni urethroplasty should be performed prior, as it has a leading cosmetic outcome with an orthotopic meatus voiding [46].

**Figure 6.**
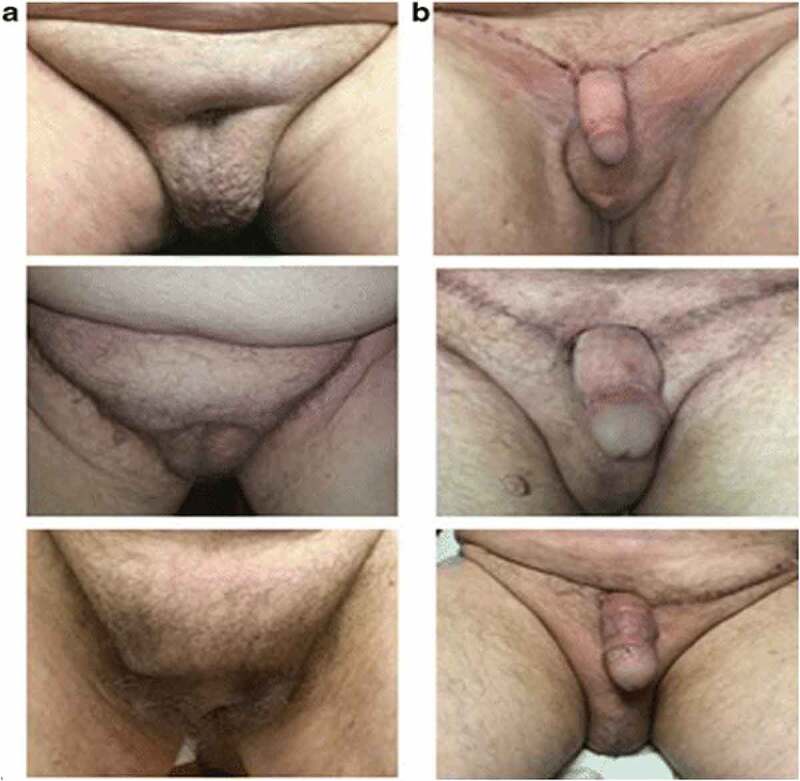
Pre- (left, a) and postoperative (right, b) management of AABP. Significant cosmetic change can be seen with escutcheonectomy and STSG. (Photographs courtesy of Fuller et al. [45])

Surgical intervention involves lipectomy of the suprapubic fat pad (escutcheonectomy) and split-thickness skin grafting (STSG). An escutcheonectomy is necessary for a successful penile unburying and proper wound healing. If an inadequate escutcheonectomy is performed there is an increased likelihood of re-burying leading to recurrence of AABP [46]. Due to large penile skin defects, STSG will be needed. Using a donor site from the lateral thigh is preferable for grafting [47]. Securing the graft to Buck’s fascia in a circumference and distally to the corona will provide proper shape for the phallus. Brown et al. [48] has shown the administration of fibrin will help improve the take of the STSG. Finally, positioning of the phallus is done with an immobilised surgical bolster (Figure 6 [45]).

Overall outcomes with AABP surgery can lead to a cosmetic improvement for the patient that could possibly be linked to improved quality of life. Studies on quality of life have not been performed, but symptoms have decreased in patients. AABP presents with many difficulties, including comorbidities that are frequently associated with obesity. Successful surgical innervation can decrease likelihood of the recurrence of AABP, which could be linked to improved quality of life. Further studies on quality of life would provide insight on the psychological impact of AABP. In conclusion, patients who are diagnosed with AABP should be referred for a urological consultation.

## Discussion

The methods and techniques encompassing penile reconstruction have steadily continued to evolve over the years. Regardless of the technique used, all methods strive to attain a common goal, which is the construction of a cosmetically appealing phallus, which allows the patient to both void standing up and to partake in penetrative intercourse that maintains adequate rigidity.

Regarding the treatment of PD, surgery was typically undertaken only in cases where curvature was extreme and symptomatic. To date, the only medically approved therapy is the use of CCh injections [1]. This paper focusses on the surgical corrective measures, although it is worth noting that treatment with CCh is often sought out first due to its non-invasive nature. In fact, in a 2017 survey, only 18% of men chose surgery at any point throughout their disease course [49]. Overall efficacy for CCh and penile plication surgery varied, with estimated rates reported at 49.5% and 79–100%, respectively [1,49].

The literature on penile transplant is sparse. Results are quite varied, and while promising, transplant remains to be an option of last resort for a multitude of reasons, such as the need for multiple operations, potential of organ rejection, and suboptimal cosmetic results [19]. Beyond the surgical complexity remains the psychological aspect of penile transplant. The penis is unlike a traditional organ transplant in a sense that it can be physically touched, making it difficult, subconsciously, for the recipient to accept the graft as their own body. Psychological guidance for both the recipient and their sexual partner should be implemented early in the transplantation process as ample time is required to fully adjust to the penile transplantation [19,20].

The RFFF has long been the gold-standard technique for phalloplasty, though ALTF has gained traction due to its cosmetically appealing results regarding the donor site. There is a bit of a trade-off here as, although ALTF produces a more concealed scar, the actual reconstruction can result in a more disproportionate phallus due to the anatomy of the donor site. Regardless of methods, both RFFF and ALTF result in good erogenous sensation. The main drawback lies in the urinary complications, particularly strictures and fistulae, that often necessitate a second corrective surgery [31]. Therefore, ensuring the patient is fully informed of the potential complications and, should one arise, high likelihood of an additional surgery is of utmost importance.

Treatment of AABP not only improves urinary and sexual dysfunction, but it also leads to significant improvements in physiological well-being. Due to the association of AABP with obesity, one can logically expect an increase in surgical repair with the rising obesity epidemic. The frequent co-occurrence of urethral strictures should be met with the goal of up-front screening for stricture disease prior to corrective surgery [43].

## Conclusion

The field of adult penile reconstruction is performed for a plethora of reasons. From cosmetic to urgent and from routine to complex, it is most definitely a growing subset of Urology, serving all men in need of it. We included more common procedures and also some of the most complex surgeries ever attempted. Our present review is the most up-to-date review of adult penile reconstruction that we know of, and we hope it will serve the literature to all those who would like a broader scope on the subject.
